# Genome rearrangements in *Escherichia coli* during de novo acquisition of resistance to a single antibiotic or two antibiotics successively

**DOI:** 10.1186/s12864-018-5353-y

**Published:** 2018-12-27

**Authors:** Marloes Hoeksema, Martijs J. Jonker, Keshia Bel, Stanley Brul, Benno H. ter Kuile

**Affiliations:** 1 Laboratory for Molecular Biology and Microbial Food Safety, Swammerdam Institute for Life Sciences, University of Amsterdam, Amsterdam, The Netherlands; 2 RNA Biology & Applied Bioinformatics, Swammerdam Institute for Life Sciences, University of Amsterdam, Amsterdam, The Netherlands; 3 Netherlands Food and Consumer Product Safety Authority, Office for Risk Assessment, Utrecht, The Netherlands

**Keywords:** Genome rearrangement, *de novo* resistance, Gene amplification, Prophage

## Abstract

**Background:**

The ability of bacteria to acquire resistance to antibiotics relies to a large extent on their capacity for genome modification. Prokaryotic genomes are highly plastic and can utilize horizontal gene transfer, point mutations, and gene deletions or amplifications to realize genome expansion and rearrangements. The contribution of point mutations to de novo acquisition of antibiotic resistance is well-established. In this study, the internal genome rearrangement of *Escherichia coli* during to de novo acquisition of antibiotic resistance was investigated using whole-genome sequencing.

**Results:**

Cells were made resistant to one of the four antibiotics and subsequently to one of the three remaining. This way the initial genetic rearrangements could be documented together with the effects of an altered genetic background on subsequent development of resistance. A DNA fragment including *ampC* was amplified by a factor sometimes exceeding 100 as a result of exposure to amoxicillin. Excision of prophage e14 was observed in many samples with a double exposure history, but not in cells exposed to a single antibiotic, indicating that the activation of the SOS stress response alone, normally the trigger for excision, was not sufficient to cause excision of prophage e14. Partial deletion of *clpS* and *clpA* occurred in strains exposed to enrofloxacin and tetracycline. Other deletions were observed in some strains, but not in replicates with the exact same exposure history. Various insertion sequence transpositions correlated with exposure to specific antibiotics.

**Conclusions:**

Many of the genome rearrangements have not been reported before to occur during resistance development. The observed correlation between genome rearrangements and specific antibiotic pressure, as well as their presence in independent replicates indicates that these events do not occur randomly. Taken together, the observed genome rearrangements illustrate the plasticity of the *E. coli* genome when exposed to antibiotic stress.

**Electronic supplementary material:**

The online version of this article (10.1186/s12864-018-5353-y) contains supplementary material, which is available to authorized users.

## Background

The ability of bacteria to acquire resistance to antibiotics relies to a large extent on their capacity for genome modification, including intracellular mobility of mobile genetic elements [1]. Prokaryotic genomes can utilize horizontal gene transfer, point mutations, and gene deletions or amplifications to realize genome expansion and rearrangements [2, 3] and are considered to be highly plastic as a result.

Prokaryotic genome content can be divided into the core genome, containing all essential and house-keeping genes, supplemented by the mobilome, composed of mobile genetic elements (MGEs) [4]. MGEs can be intercellular, such as plasmids, integrative and conjugative elements (ICEs), and extracellular in the form of bacteriophages. Bacteriophages are major drivers of horizontal transfer of virulence factors [5] and antibiotic resistance genes [6]. While lytic phages ultimately induce bacterial cell lysis, lysogenic or temperate phages integrate into the bacterial genome and replicate with the host genome as prophages [7]. After integration, prophages can undergo a complex decay process involving point mutations, genome rearrangements, deletions, and invasion by other mobile DNA elements [8], resulting in cryptic prophages that are metabolically and genetically inert. In *Escherichia coli* (*E. coli*) K-12, nine cryptic prophages remain, accounting for 3.6% of all genomic DNA [9], which contribute to survival in adverse environments such as exposure to antibiotics, oxidative stress, heat stress, and acid stress [10].

Intracellular MGEs are not by themselves transmissible to other cells, but can change location within the genome. Transposons, introns, and insertion sequences (ISs) belong to this category. ISs constitute an important part of most prokaryotic and eukaryotic genomes, occurring in a wide range of copy numbers [11]. ISs, which vary in size from 0.7–2.5 bp, only carry genes involved in their transposition but can induce duplications, deletions, and genome arrangements [12]. Because of the coding density of the prokaryotic genome, most insertions are expected to cause frame-shifting and thus deleterious alterations, but some may confer a selective advantage by providing new regulatory sequences [13–15].

The contribution of point mutations to de novo acquisition of antibiotic resistance is well-established [16, 17]. Gene duplication and amplification plays an important role in creating genomic variability, enabling adaptation to modified growth conditions [18]. Gene amplification in response to antibiotic stress has been reported with duplications ranging from a few bp [19] to 300 kb [20]. Gene deletions also contribute to development of antibiotic resistance [21–23]. Several questions are unanswered at present: is de novo development of resistance accompanied by genomic rearrangements? If yes, do the same rearrangements occur during to induced resistance against different antibiotics? Do the same genetic events always occur during to exposure to the same drug?

Here, we provide an overview of genome rearrangements that occur in populations of *E. coli* cells exposed to increasing subinhibitory concentrations of amoxicillin, enrofloxacin, kanamycin, or tetracycline.

## Results

**Fig. 1:**
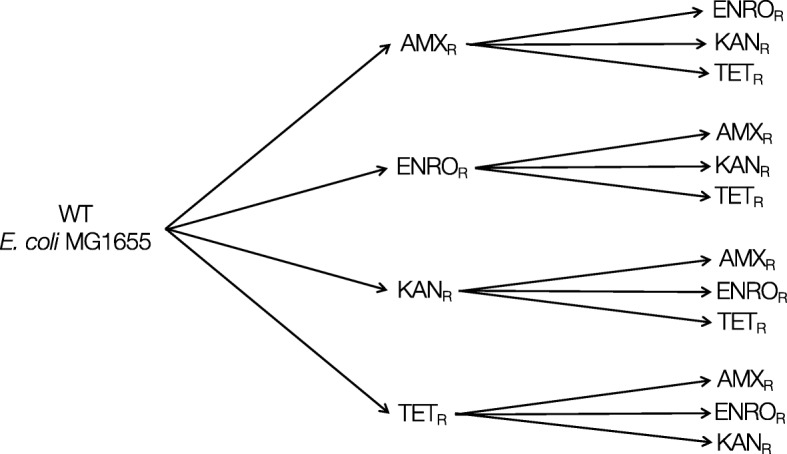
Experimental scheme. Wild-type *E. coli* MG1655 was exposed to stepwise increasing concentrations of amoxicillin (AMX), enrofloxacin (ENRO), kanamycin (KAN), or tetracycline (TET), resulting in strains with a de novo acquired resistance to a single antibiotic. The resistant strains were subsequently made resistant to the three other antibiotics. Each induction of resistance was performed in duplicate, resulting in four replicates with an identical exposure history of the double resistant strains

The main objective of this study was to investigate whether genome rearrangements occur in *E. coli* during de novo acquisition of resistance to antibiotics. Whole genome population sequencing was applied to compare wildtype *E. coli* to populations (app. 10^9^ cells) derived from that wildtype with acquired resistance to either one, or to two antibiotics sequentially [24] (Fig. 1). Genomic DNA was isolated from strains developed in four independent rounds of experiments inducing resistance to specific antibiotics by growing the cells at step-wise increasing concentrations. An overview of all identified genome rearrangements is presented in Table 1. These elements will be discussed separately below.

**Table 1:** Overview of genomic alterations observed after acquisition of resistance to amoxicillin, enrofloxacin, kanamycin, or tetracycline in wild-type (for the first exposure) or in strains with previously acquired resistance to amoxicillin (AMX_R_), enrofloxacin (ENRO_R_), kanamycin (KAN_R_), or tetracycline (TET_R_) (for the second exposure)

	+ amoxicillin				+ enrofloxacin				+ kanamycin				+ tetracycline			
	WT	ENRO_R_	KAN_R_	TET_R_	WT	AMX_R_	KAN_R_	TET_R_	WT	AMX_R_	ENRO_R_	TET_R_	WT	AMX_R_	ENRO_R_	KAN_R_
Amplifications																
*ampC*	2/2	4/4	4/4	4/4												
Deletions																
*clpS-clpA*								1/4			1/3	1/4				
*yaiT-yaiW*											1/3					
*slyA-nemA*				1/4												
*prophage e14*		2/4	2/4	1/4		1/4	2/4	1/3				1/4		4/4	3/4	
Insertions																
*fimA*	2/2	4/4	3/4	1/4		1/4				2/3				3/4		
*yeaR*						4/4	1/4	2/4			3/3				4/4	
*dcuC/pagP*											3/3	2/4				1/1
*mgrB/yobH*				3/4								3/4				1/1
*oppB*											1/3	1/4				
*clpX/lon*						2/4		1/3							1/3	
*cyoA*			1/4													

X/Y: X indicates number of strains with genomic alternation, Y indicates total number of sequenced strains. A more comprehensive table detailing which genome rearrangement was identified in which replicate is provided as supplemental information (Additional file 1: Table S1)

**Fig. 2:**
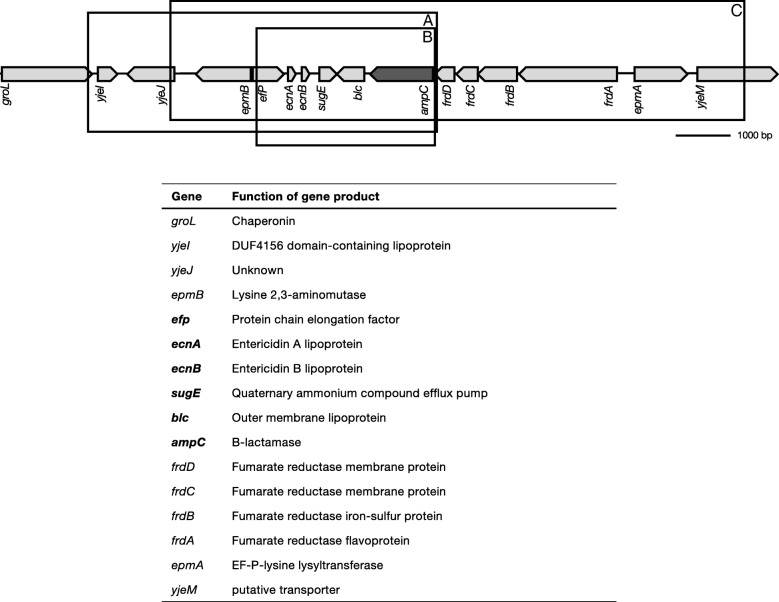
Amplification of three different fragments, all including *ampC*, upon acquisition of resistance to amoxicillin. Fragment B and C were detected in tetracycline resistant strains exposed to amoxicillin, fragment A was detected in all strains with an *ampC* amplification. See Table 1 for detailed information on prevalence of shown amplifications. The figure depicts genomic organization at point of deletion. The genes involved and the resulting gene products are displayed under the figure. Genes in bold are amplified in all three fragments

**Fig. 3:**
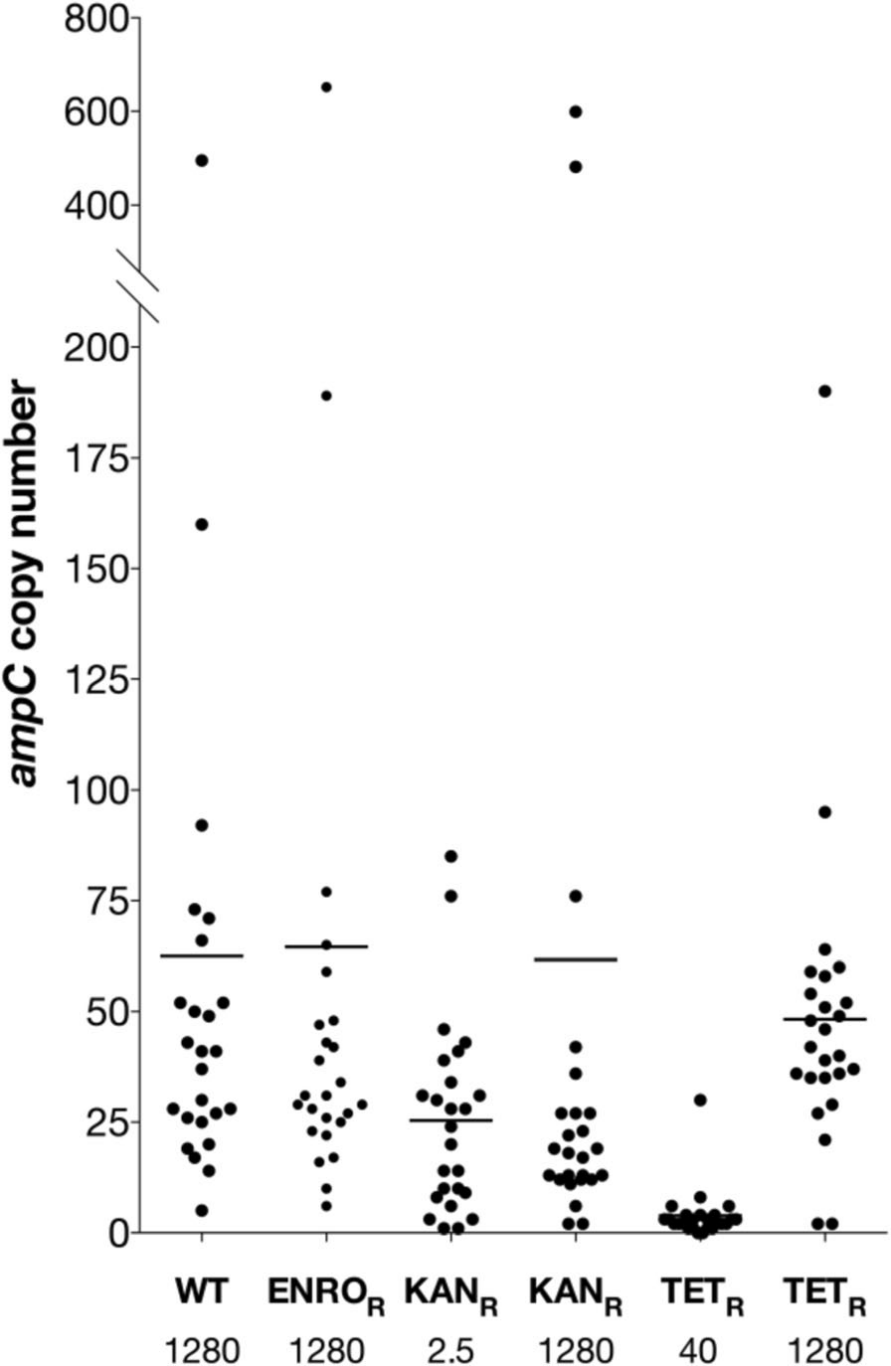
*ampC* copy number for different strains carrying an *ampC* amplification. The *ampC* copy number was determined with qPCR, using untreated wild-type *E. coli* as a reference. With exception of wild-type (which only acquired resistance to amoxicillin), all strains carried a previous resistance to enrofloxacin (ENRO_R_), kanamycin (KAN_R_), or tetracycline (TET_R_), resulting in a secondary resistance to amoxicillin The number displayed under the strain indicates the concentration amoxicillin used for resistance development. Bars indicate the average copy number from 25 colonies

In all cells with acquired resistance to amoxicillin, either primary or secondary, (Table 1), *ampC* was amplified (Fig. 2). Three amplicons were identified varying in size from 3.5 to 10.5 kb (Fig. 2, a-c). Amplicon B-C were present in tetracycline resistant cells exposed to amoxicillin, all other cells contained amplicon A. In addition to *ampC*, 9 other genes were present in all three amplicons. Because population sequencing only provides the average copy number for the entire population, qPCR was used to quantify the number of repeats for one set of evolution experiments (Fig. 3). Strains with low levels of induced amoxicillin resistance carry on average 3–25 copies of the *ampC* gene. In strains that developed resistance to 1280 μg/mL amoxicillin, the average copy number ranges from 48 to 65. Within single populations, the *ampC* copy number varied strongly, with copy numbers ranging from single digits to a few hundred, suggesting high amplicon instability.

**Fig. 4:**
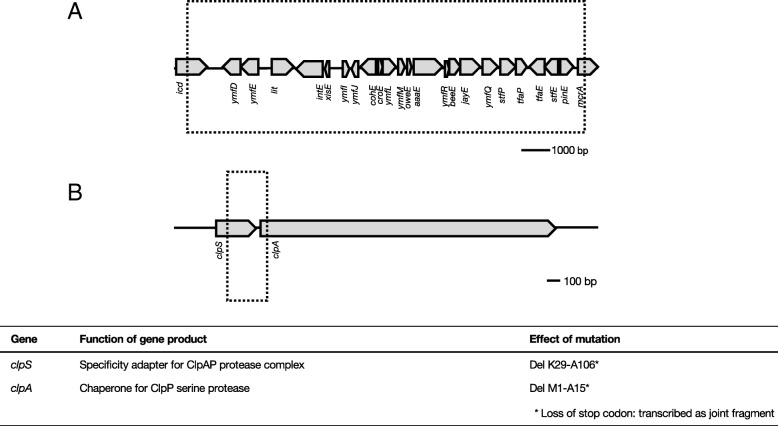
Deletions detected in strains with de novo acquired antibiotic resistance **a**: Deletion of prophage e14 associated genes in strains exposed to any of the four antibiotics. **b**: Partial deletion of *clpS* and *clpA* in strains exposed to enrofloxacin and tetracycline. Figures depict genomic organization at point of deletion. The genes involved and the resulting gene products are displayed under the figure. Prophage associated genes are not shown because the resulting gene products are mostly not characterized. See Table 1 for detailed information on prevalence of the deletions

In addition to the *ampC* amplification, deletions were also identified in various strains. A 14.4 kb deletion (Fig. 4a) was detected in 17 samples. No correlation with exposure to a specific antibiotic could be identified, but excision only occurred when cells were exposed to a second antibiotic (Table 1). All deleted genes were identified as part of prophage e14. A 312 bp deletion in *clpS* and *clpA* (Fig. 4b) was identified in one of the tetracycline resistant strains exposed to enrofloxacin (Table 1). The reading frame is not disturbed, but the 312 bp deletion includes the *clpS* stop codon and the *clpA* start codon, resulting in a fusion protein containing 28 N-terminal amino acids from clpS and 743 C-terminal amino acids from clpA.

**Fig. 5:**
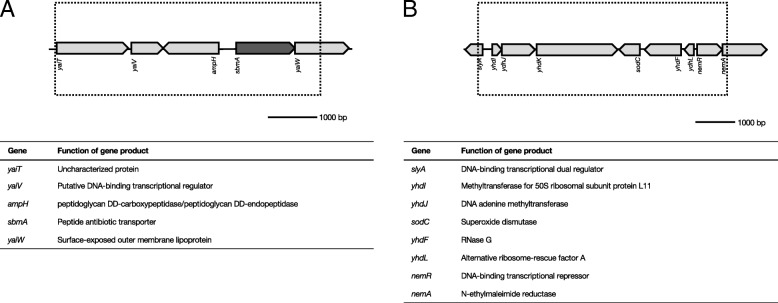
Deletions detected in resistant strains made resistant to kanamycin and amoxicillin. **a**: Deletion of *sbmA* and surrounding genes in enrofloxacin resistant *E. coli* exposed to kanamycin. **b**: Partial or full deletion of 8 genes upon induction of amoxicillin resistance in tetracycline resistant *E. coli*. Figures depicts genomic organization at point of deletion. The genes involved and the resulting gene products are displayed under the figure. See Table 1 for detailed information on prevalence of the deletions

In a de novo enrofloxacin resistant *E. coli* subsequently treated with kanamycin, a 5.4 kb deletion was observed, containing 4 full-length and one partial gene deletion (Fig. 5a). Most notably, *sbmA*, encoding the peptide antibiotic transporter associated with kanamycin resistance, is located within this deletion. Point mutations in this gene were also found in many strains with acquired resistance to kanamycin (accompanying article). A 6.1 kb deletion, composed of 7 full-length and 2 truncated genes, was detected in a strain exposed to tetracycline after acquisition of resistance to amoxicillin (Fig. 5b). Two partially deleted genes, *slyA* and *nemA*, as well as 6 full-length genes are included in this deletion.

**Fig. 6:**
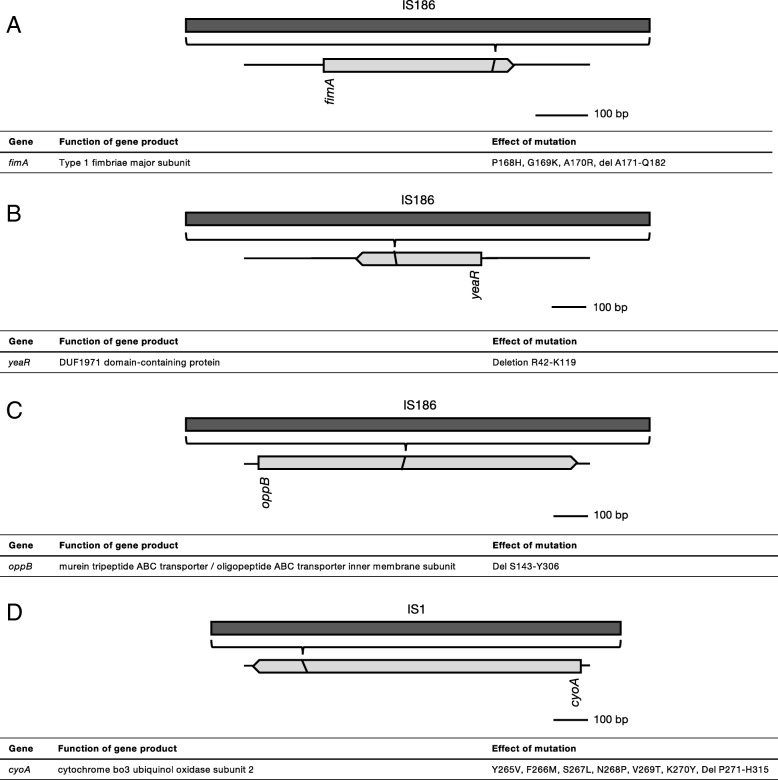
Intragenic IS transpositions identified in strains with acquired antibiotic resistance. IS186 insertion was detected in *fimA* in cells with acquired amoxicillin resistance (**a**), in *yeaR* in cells with acquired amoxicillin resistance (**b**), and in *oppB* in cells with secondary kanamycin resistance (**c**). IS1 insertion was found in *cyoA* in a single kanamycin resistant strain exposed to amoxicillin (**d**). See Table 1 for detailed information on prevalence of shown IS transpositions

In addition to genome amplifications and deletions, the role of transposable elements in acquisition of antibiotic resistance was also investigated. Two different insertion sequences, IS*186* and IS*1*, were detected in four different genes (Fig. 6). Insertion of IS*186* in *fimA* and *yeaR* correlates with exposure to amoxicillin or enrofloxacin, respectively (Table 1). Transposition of IS*186* into *oppB* or IS1 into *cyoA* is much rarer, in comparison. In all four cases, the reading frame is disturbed, resulting in C-terminal deletions.

**Fig. 7:**
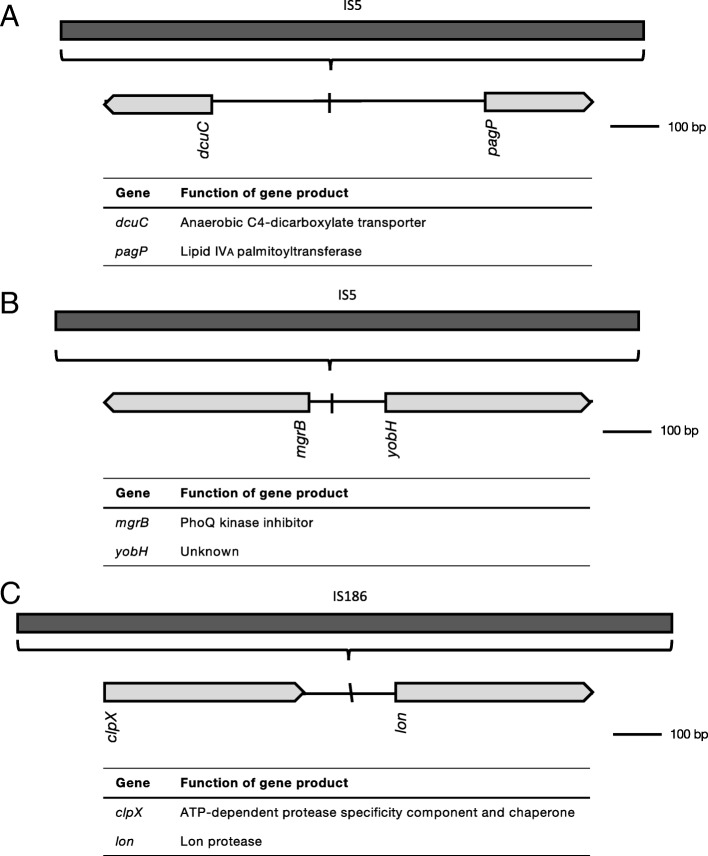
Intergenic IS transpositions identified in strains with acquired antibiotic resistance. IS5 was found in the 5’ UTR of *dcuC* and *pagP* when cells were exposed to kanamycin (**a**), and *mgrB* and *yobH* upon acquisition of resistance to tetracycline (**b**). IS186 transposition was detected in the 5’ UTR of *lon* in enrofloxacin resistant cells exposed to tetracycline (**c**)

Along with intragenic, intergenic transposition of IS*5* into the 5’ UTR of *dcuC* and *pagP*, and the 5’ UTR of *mgrB* and *yobH* was detected as well (Fig. 7a/b). IS*5* insertion into the 5’ UTR of *dcuC* and *pagP* was associated with kanamycin resistance, while transposition of IS*5* into the 5’ UTR of *mgrB* and *yobH* only occurred upon exposure to tetracycline (Table 1). Finally, IS*186* was detected in the 5’ UTR of *lon (*Fig. 7c), most likely associated with exposure to enrofloxacin (Table 1).

## Discussion

In *E. coli*, genome plasticity is a main source of functional diversity on a genomic level, enabling adaptation to diverse environments. Here, we show that several genome rearrangements occur when *E. coli* acquires resistance to different antibiotics. The data presented here, combined with information available on point mutations acquired during development of resistance (accompanying article), suggest that the organism uses several strategies to deal with antibiotic stress. In contrast, *Pseudomonas aeruginosa* exposed to a similar regimen of increasing antibiotic concentrations, only acquired point mutations [17], highlighting the ability of *E. coli* to adapt to antibiotic stress using several different approaches.

Gene amplification in response to antibiotic treatment has been reported before, and can result in antibiotic resistance through overproduction of target molecules [20, 25], efflux pumps [26], target modification [27], or antibiotic-modifying enzymes such as B-lactamases [28]. The level of chromosomal B-lactamase, and therefore the level of amoxicillin resistance, is gene-dose dependent [29]. Moreover, promoter mutations can result in a 6 to 21-fold increase in promoter strength [30, 31]. A wild-type strain adapted to 40 μg/ml does not yet carry any *ampC* repeats (data not shown) but does contain promoter mutations (accompanying article), indicating that intermediate level resistance does not require additional gene copies. In contrast, B-lactam resistance in *Salmonella typhimurium* is initiated by beta-lactamase gene amplification, followed by stabilizing point mutations [32].

Considering the size of the *E. coli* genome and the amplicon size, the number of amplifications carried by a single cell (Fig. 3) implies that, on average, strains with high levels of amoxicillin resistance increase their genome size by 5–10%. The cost of carrying amplifications has been shown to be determined mainly by the metabolic costs of the encoded enzyme rather than the cost of synthesizing additional DNA [33]. The resulting increased protein activity is therefore likely to require some kind of compensation. No difference in maintenance energy between wild-type and amoxicillin-adapted *E. coli* was detected, but amoxicillin-resistant *E. coli* showed a narrowing of the ecological range in the form of reduced pH- and salt-tolerance [34].

Gene amplifications are considered to be intrinsically unstable as homologous recombination can occur between identical repeats [18]. Beta-lactamases are secreted into the periplasm [35], hence cells that do not produce any beta-lactamase can still be protected by the enzymes produced by neighboring cells [36, 37]. Together with the metabolic costs of producing enzyme, these factors could be driving the loss of copies and explain the observed variation in copy number (Fig. 3).

Most of the genome rearrangements observed only occur in strains with a secondary acquired resistance, and not during primary exposure (Table 1). This includes the deletion of cryptic prophage e14 (Fig. 7), and transposition of insertion sequences (Figs. 6 and 7). Prophage e14 is excised after induction of the SOS response [38] and has been shown to follow norfloxacin exposure [39]. Likewise, IS transposition has been shown to occur after activation of the SOS response [40]. Although SOS response activation has been reported to follow exposure to beta-lactams [41, 42] and quinolones [43, 44], we do not observe the expected genome rearrangements during primary exposure. This suggests that, in our experimental conditions, either SOS response activation is not enough to trigger excision or transposition, or the SOS response itself is not sufficiently activated.

Prophages, although remnants of defective phages, are recognized to be functional during bacterial stress [45]. Exposure of *E. coli* to nalidixic acid or azlocillin results in induction of expression of the prophage e14 genes *ymfL* and *ymfM* [10], both hypothesized to be cell division inhibitors [46]. Furthermore, single deletions of either *ymfL* or *ymfM* result in a reduced ability to resist oxidative stress. As reactive oxygen species (ROS) production in antibiotic resistant cells exposed to other antibiotics is lowered [24], excision of this prophage is in line with the radical-based theory, which suggests a pivotal role for ROS in the action of bactericidal antibiotics.

Insertion sequences are necessary for mediating large-scale variation during bacterial genome evolution [47]. The *E. coli* genome contains many insertion sequences, among which IS5 and IS186 are considered to be among the most active [48]. The point of insertion can be specifically correlated with resistance to one antibiotic; *fimA* for amoxicillin resistance, *yeaR* and *lon* for enrofloxacin resistance, *dcuC*/*pagP* for kanamycin resistance, and *mgrB*/*yobH* for tetracycline resistance, indicating that IS transposition is not a random event (Table 1). As a single IS element can integrate in many genomic locations [11], the observed insertions likely contribute to resistance development.

Intragenic insertion of an insertion sequence most often results in a loss of function of the resulting gene product [49, 50]. In this dataset, intragenic insertion was observed in four different genes, including *fimA* (Fig. 6a). *fimA* codes for a type-1 fimbrial protein, which is a virulence factor in pathogenic *E. coli* [51]. Resistance to quinolones is associated with a decrease in *fimA* expression, caused by an IS10 transposition into *fimA* [52]. In general, antibiotic resistance is correlated with lowered virulence [53, 54], but such association has yet to be established for beta-lactam resistance and *fimA* expression.

IS*186* has previously been described to cause fluoroquinolone resistance by inserting in the coding sequence of the AcrAB repressor *acrR* [55]. In our data set, this insertion was not observed, but rather a transposition of IS*186* into *yeaR* (Fig. 6b). Although *yeaR* expression is induced in response to nitrate and nitrite [56], or nitric oxide [57], the function of the resulting gene product is as of yet unknown.

In *Pseudomonas aeruginosa*, *oppB* is involved in pacidamycin resistance [58]. In *E. coli*, it is required for uptake of phaseolotoxin, but currently there is no evidence for a role in antibiotic resistance [59]. Likewise, for *cyoA* there is no known connection to development of antibiotic resistance. In addition, transposition of IS*1* into *cyoA* only occurred in a single replicate and might therefore be less relevant (Fig. 6d).

Intergenic insertions may disrupt promoter function or create new promoters, thereby modifying gene expression, which has been observed in antibiotic-resistant bacterial strains [13, 55, 60–62]. Intergenic insertion of IS*5* or IS*186* took place on three different occasions. Insertion of IS*5* in the 5’ UTR of *dcuC* and *pagP* (Fig. 7a) is exclusively associated with kanamycin exposure. Neither *dcuC*, responsible for the transport of C4-dicarboxylates during anaerobic growth, nor *pagP*, a lipid A palmitoyltransferase, are known targets involved in resistance to aminoglycosides. However, as aminoglycosides bind to the outer membrane during entry into the bacterial cell [63], alteration of the lipid A structure might result in a decreased affinity of aminoglycosides for the membrane. LPS changes in the outer membrane have been linked to aminoglycoside resistance [64]. In *Salmonella typhimurium*, deletion of *pagP* results in hypersensitivity to antimicrobial peptides [65].

Another IS*5* transposition upstream of *mgrB* and *yobH* (Fig. 7b) is correlated with resistance to tetracycline (Table 1). *MgrB* negatively regulates the two-component system PhoP/PhoQ, which controls virulence and adaptation to Mg^2+^-limited environments [66]. Insertions of IS*5* family elements within *mgrB* have been shown to cause polymyxin resistance [67, 68], but no information exists on the contribution of this element to tetracycline resistance. The role of *lon* during development of antibiotic resistance is well-established. IS*186* insertions into the *lon* promoter have been reported before [69, 70] and contribute to low level multidrug resistance through stabilization of Lon protease substrates MarA and SoxS [71].

In general, no correlation can be found between the presence of different rearrangements as different combinations are observed in many strains (Additional file 1: Table S1). The number of genome rearrangements detected varies from 1 to 4 per sequenced strains, and this does not appear influenced by the number of acquired point mutations (accompanying article). The appearance of the same rearrangement in independent lineages is most likely a reflection of the specificity of the response to different antibiotics. Although genetic drift cannot be excluded as a driver, it is not very likely as a wildtype control after even more cell duplications had only 6 point mutations and no other modifications.

## Conclusions

In general, the overview of all genomic alterations presented here illustrates the remarkable plasticity of the *E. coli* genome when exposed to antibiotic stress. Many of the amplifications, deletions, or insertions have not been reported before as genomic modifications occurring during resistance development. However, the appearance in all or at least several replicates indicates that these events are not likely to occur randomly and hence might play a functional role during acquisition of antibiotic resistance.

## Materials and methods

### Sample description

All samples for sequencing were gathered from experiments described in [24]. Briefly, batch cultures of wild-type *E. coli* were adapted to increasing concentrations amoxicillin, enrofloxacin, kanamycin, or tetracycline, followed by a second round of adaptation to any of the three other antibiotics (Fig. 1). For every step, bacteria were reinoculated to an OD_600_ of 0.1. Each round of adaptation was performed twice, resulting in four secondary rounds of adaptation for each antibiotic. This way four strains derived from the same wildtype with an identical exposure history were obtained.

### WGS

Genome isolation was carried out with the DNeasy blood and tissue kit (Qiagen). Samples were prepared for IonTorrent sequencing as described before (accompanying article). After the quality control and read mapping, the BAM files were subjected to copy number analysis using the cn.mops package in R (https://cran.r-project.org/) [72].

The copy number analysis procedure entailed: 1) segmentation of the genome in counting bins, 2) counting the reads for each bin, 3) sample normalization and GC correction, and 4) copy number detection in each sample. *Loci* with amplifications or deletions indicated by a ≥ 2-fold difference in copy number were selected. All genomic aberrations detected by the algorithm were checked by visual inspection of the data at each particular genomic region. In addition, stretches of single nucleotide polymorphisms identified in the TVC-generated data were found to be indicative for a suboptimal mapping result due to insertions. Insertions detected in this way were confirmed with PCR or qPCR. No genome rearrangements were detected in the sequenced wild-type strain. Deletions smaller or equal to 26 nucleotides were described in the accompanying paper.

### PCR

PCR was used to verify a number of amplifications, deletions, or insertions. Primers are given in Table 2. Amplification was performed in 25 μL working volumes with DreamTaq polymerase (Thermo Scientific) with the following cycling conditions: 5′ at 95 °C, 35 cycles of 35″ at 95 °C, 55″ at given annealing temperature and 90″ at 72 °C, ending with a 90″ extension at 72 °C. PCR products were purified using the MSBSpinRapace kit (Stratec) and sequenced by Macrogen Europe using Sanger sequencing.

**Table 2:** Primers used for PCR

Gene	Sequence (5′ - > 3′)	Annealing temperature (°C)
*clpS-clpA*	fw TGTGACAGATGTCGCTGATG	49
	rv AAAGGCTTCCAGTTCCTGAC	
*fimA*	fw AGCTGAATGATTGCGATACCA	49
	rv GAAACCGGTTACTGCTGATTTG	
*yeaR*	fw GAACGTACGGTATTCACCAGAT	49
	rv GAACGTACGGTATTCACCAGAT	
*dcuC-pagP*	fw GCGAGCTACACCCACAATAA	49
	rv GTCATCCACTCATCTGCGTTAG	
*mgrB-yobH*	fw GAAGAACCACCACCGATACAA	49
	rv CGCCATATCCGCTGAGTAATAA	
*clpX-lon*	fw GTTGAATGAACTGAGCGAAGAAG	56
	rv TGCGCGACCAGCATAAT	
*oppB*	fw CCAGAAGGTAGGGCAATGTT	56
	rv CAATCATAGAGCCACGGGTAAT	
*cyoA*	fw TAATGCCAGCGATCGTAACC	56
	rv CAACTCCGTGATGAACTCCTT	

### Quantitative PCR

Single colonies were dissolved in 10 μL TE-buffer (pH 8.0) and incubated at 95 °C for 5 min, after which the sample was diluted 10^5^ fold in sterile MilliQ. 5 μL of diluted sample was mixed with 20 μL master mix containing 50 nM of each primer and Power SYBR Green PCR mix (Thermofisher Scientific). Quantitative PCR was performed with the Applied Biosystems 7300 real-time PCR system (Applied Biosystems) using the following cycling conditions: 10′ at 95 °C, 40 cycles of 15″ at 95 °C and 1′ at 60 °C. A wild-type sample was prepared as described above and aliquoted for use as a reference on every plate. Cycle threshold (Ct) values were determined by automated threshold analysis using the ABI Prism 1.0 software. Gene copy numbers were determined using the ∆∆Ct method using *idnT* as the reference gene. *IdnT* was chosen because no mutations or other alterations were detected in this region for any of the resistant strains. Primers used for quantification are shown in Table 3 and were validated using serial dilutions of WT sample.

**Table 3:** Primers used for quantitative PCR

Gene	Sequence (5′ – 3′)
*idnT*	fw CGCCACTACGCTGATTGCT
	rv TCACTAGCGCCCATTGCA
*ampC*	fw CGATACTGGAGTTGGCATACAG
	rv GACTTGCTGCGCTTCTATCA

## Additional file

Additional file 1: **Table S1.** Overview of genomic rearrangement occurring in different samples. Contains information on the combinations of genome rearrangements that appear in samples, as well as correlation with the number of point mutations also identified. (XLSX 19 kb)

## Abbreviations

Amx: Amoxicillin
Enro: Enrofloxacin
ICE: Integrative and conjugative elements
IS: Insertion sequence
Kan: Kanamycin
MGE: Mobile genetic element
ROS: Reactive oxygen species
Tet: Tetracycline
